# Prevalence, Determinants, and Effects of Food Insecurity among Middle Eastern and North African Migrants and Refugees in High-Income Countries: A Systematic Review

**DOI:** 10.3390/ijerph17197262

**Published:** 2020-10-04

**Authors:** Reima Mansour, Pranee Liamputtong, Amit Arora

**Affiliations:** 1 School of Health Sciences, Western Sydney University, Campbelltown Campus, Penrith, NSW 2751, Australia; p.liamputtong@westernsydney.edu.au (P.L.); a.arora@westernsydney.edu.au (A.A.); 2 Translational Health Research Institute, Western Sydney University, Penrith, NSW 2751, Australia; 3 Discipline of Child and Adolescent Health, Sydney Medical School, Faculty of Medicine and Health, The University of Sydney, Westmead, NSW 2145, Australia; 4 Oral Health Services, Sydney Local Health District and Sydney Dental Hospital, NSW Health, Surry Hills, NSW 2010, Australia

**Keywords:** food security, food insecurity, Middle Eastern, North African, MENA, migrants, refugees

## Abstract

Issues related to poverty and income inequality in high-income countries have led to food insecurity among some population groups, such as migrants and refugees. While there are some studies on the experience of some migrant groups (and other subpopulations), little is known about food security among Middle Eastern and African migrants and refugees. This systematic review identified the prevalence of food insecurity and its effects among Middle Eastern and North African (MENA) migrants and refugees in high-income countries. The Preferred Reporting Items for Systematic Reviews and Meta-Analyses (PRISMA) guidelines were followed in this systematic review. Four databases, namely MEDLINE (Ovid), Embase (Ovid), CINAHL (EBSCO), and PubMed were searched. Three studies met the inclusion criteria, all of which were conducted in USA: two among Sudanese migrant families, and one among Somali refugee women. The rates of reported food insecurity ranged from 40% to 71% and were significantly higher than for the general population. Food insecurity was associated with acculturation and socio-economic factors. Food insecurity adversely impacts the health of MENA migrants and refugees, creating economic implications for individuals, families, the broader community in which they now live, and for governments.

## 1. Introduction

Every individual has the right to adequate food and to be free from hunger regardless of their socio-economic or socio-cultural status, as proclaimed in 1948 in the United Nations (UN) Universal Declaration of Human Rights (UDHR) and reiterated in the International Covenant on Economic, Social and Cultural Rights (ICESCR) [1]. This right implies that food is to be available not just in sufficient quantity and quality (and safe), but also “acceptable within a given culture”, and access to it should be sustainable and not interfere with other human rights [1]. This right is clearly linked to food security in many groups of people, including migrants and refugees.

External and internal conflicts, together with natural disasters, have generated vast numbers of internally and internationally displaced persons [2]. These populations, whether in their home countries or dispersed internationally as refugees, present challenges to the international community and host countries in terms of meeting their nutritional needs. There are also migrants able to choose to move for education or temporary employment or able to emigrate and secure permanent residency. The number of people ‘on the move’ as migrants and refugees is currently at its highest level since the period immediately after World War II [3]. Over the past two decades, the number of international migrants globally (including refugees who comprise about 10% of international migrants) has increased from 174 million in 2000 to 272 million in 2019 [4] (prior to COVID-19). 

Due to the rising number of migrants and refugees, food security among these groups has become an increasing concern for international aid agencies and host country governments. Although more than half of all international migrants worldwide are hosted in high-income countries (HICs) [4], a substantial proportion of refugees are hosted in low- or middle-income countries, such as Turkey, Uganda, Palestine, and Pakistan [3]. Those refugees who do eventually settle in HICs (such as the USA, UK, and Australia) can still face problems in terms of food security, including nutrition [5,6]. Research among migrant/refugee populations to discern the reasons for continued food insecurity for these populations in HICs is a necessary prelude to addressing any problem effectively. It is this area that this systematic review explores.

Among migrant and refugee populations, the factors that have been implicated in food security include: language barriers, culturally determined dietary preferences which may remain unsatisfied in the new country, and a lack of familiarity with nutritionally sound substitutes [5]. Language barriers and difficulties with adaptation to a new cultural environment (including foods) are common to both migrants and refugees [6,7] and are associated with food insecurity. The culture of migrants/refugees also has a great impact on their choice of foods [8]. People from different cultural backgrounds have different food patterns and preferences, and access to traditional foods may be of importance for identity, nutrition, health, and cultural reasons [9]. Researchers have found that migrants/refugees often consume traditional food as a way of retaining their cultural identity [10,11,12,13]. In the absence of familiar foods, however, migrants may be less able to make nutritionally optimal choices [5], especially in the context of language difficulties. 

The Middle East and North Africa cover an extensive geographic region stretching from Morocco to Iran, involving 20 countries [14]. Since the 1960s, the ongoing conflicts and a variety of divisions in MENA have significantly altered the stability in the region [14,15], leading to a situation where a substantial proportion of refugees all over the world being from MENA countries. The UN High Commissioner for Refugees (UNHCR) states that about two-thirds of refugees (67%) come from Syria (6.7 m), Afghanistan (2.7 m), South Sudan (2.3 m), Myanmar (1.1 m), Somalia (900,000), Sudan (725,000), and the Democratic Republic of the Congo (720,300) [14]. That five of the seven major countries are Middle Eastern (ME) and African countries highlights the political instability in the Middle East and North Africa that triggers mass displacement [14]. 

Refugee status, especially when combined with a different cultural and linguistic background, has also been known to be associated with food insecurity [6,13]. The MENA region has historically been a crossroads of different cultures and religions (Judaism, Christianity, and Islam), resulting in specific religiously restricted dietary requirements, such as for Halal and Kosher food [16]. Studies conducted in lower-income host countries (such as Turkey, Lebanon, Jordan, Syria) which accommodate the larger proportion of refugees have found food insecurity to be a major concern among ME refugees and revealed a need for improved food and financial assistance to these vulnerable populations [17,18,19]. Food insecurity can also be high among some refugee populations in their countries of origin (e.g., South Sudan [20]), which can have enduring impacts that range from developmental delays to physical and mental health concerns in refugees who have experienced such deprivation. 

Food insecurity and nutritional inadequacy among refugees have been recorded in a number of studies, including in the UK [21] and Australia [6], as well as in the USA [22,23] and Canada [24,25]. However, studies involving migrants/refugees from MENA countries were relatively few compared to those involving older immigrant populations or populations from other regions in the world (e.g., Liberians [22], and Cambodians and Brazilians [23] in the USA, and Latinos in Canada [24]). According to Asbu et al. [15], there are gaps in knowledge about the health status of MENA migrants who have recently arrived in HICs. It is only in comparatively recent times that researchers have sought to investigate experiences of the migrant and refugee arrivals in terms of food insecurity, nutrition, and health [6]. Such efforts, however, have often been marred by limitations in terms of sample size and composition as well as a tendency to encompass a mix of cultural and ethnic backgrounds, and hence a failure to focus solely on MENA refugees and/or migrants. This review focused on MENA migrants and refugees as they are amongst the most recently arrived groups, and little is known about them in the literature. While some comparisons may be drawn with other more general studies, the scope of this review was to explore the experiences of MENA migrants and refugees.

In order to address this issue fully and establish the level of evidence that has been conducted in this area, this review aims to determine the prevalence, determinants, and effects of food insecurity among MENA migrants and refugees in HICs. 

## 2. Materials and Methods 

The review followed the Preferred Reporting Items for Systematic Reviews and Meta-Analyses (PRISMA) guidelines [26] (see Appendix A for the complete PRISMA checklist). 

### 2.1. Eligibility Criteria

The review utilized a non-intervention interrogation of existing research using the Sample (S), Phenomenon of Interest (PI), Design (D), Evaluation (E), Research type (R) (SPIDER) tools to define the eligibility criteria [27]. The eligibility criteria have been selected in accordance with the SPIDER tool [27]. The sample (S) comprised studies investigating the status of food security/insecurity and challenges related to access to affordable foods that meet cultural needs. The Phenomenon of Interest (PI) was Middle Eastern and/or North African migrants or refugees residing in high-income countries. No specific study design (D) was selected so as not to limit the search findings. Views and experiences of the participant group, not the researchers, formed the basis for study selection (E). Both qualitative and quantitative research types (R) were included (see Table 1).

### 2.2. Information Sources

In order to gain a full collection of articles that reported on food security research in high-income countries, no limits were placed on publication dates, age, gender, or language. The following four key nutrition and health sciences electronic databases were searched—Medline (OVID), Embase (OVID), PubMed, CINAHL (EBSCO). Additionally, a manual search of the reference lists of all the eligible studies detected and of previously published systematic reviews was performed. Furthermore, the professional and publication profiles of authors of included studies were also searched to increase the possibility of capturing all relevant research evidence. The search strategy aimed to locate only published studies. The search was initially conducted in October 2018 and then updated in December 2019 to obtain any additional studies that might have been published. A final search was conducted in September 2020 to keep the search updated and current. 

### 2.3. Search Strategy

The current study used the Sample, Phenomenon of Interest, Design, Evaluation, Research type (SPIDER) tool [27] to devise the review question and related search terms. A combination of specified subject headings (MeSH) terms and keywords was drafted in collaboration with expert Medical and Health Sciences librarians. A sample search was conducted using CINAHL (see Appendix B). This study used various combinations of the subject heading terms and keywords: food security; food insecurity; food access; food preferences; food availability; food utilization; food stability; Middle Eastern; North African; immigrants; migrants; refugees; displaced persons; resettled. The search strategy was pre-tested in the CINAHL (EBSCO) database and subsequently adapted to the syntax and subject headings of three other databases (MEDLINE (Ovid), Embase (Ovid), PubMed). 

### 2.4. Study Selection

**Figure 1:**
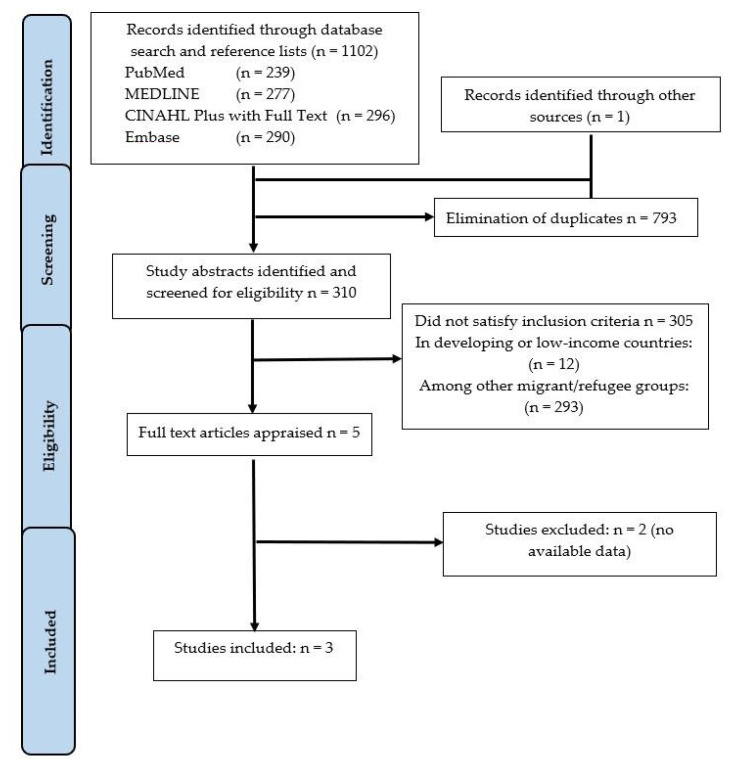
Preferred Reporting Items for Systematic Reviews and Meta-Analyses flow chart of the literature search and study selection process.

Studies identified through the electronic databases and manual search were exported into a reference manager software Endnote X9 (Clarivate analytics, London, United Kingdom,) for removing duplicates, screening, and selection. Two reviewers (Reima Mansour and Amit Arora) independently screened the articles based on the eligibility criteria. The excluded studies and the specific reasons for exclusion as they did not meet the inclusion criteria are recorded in Appendix C. The study selection process has been presented as a PRISMA flow chart (see Figure 1). 

### 2.5. Data Collection Process and Data Items

Data from the included studies were extracted independently and in duplicate by two reviewers (Reima Mansour and Amit Arora), and any subsequent disagreements were resolved through discussion with a third reviewer (Pranee Liamputtong). The information extracted from included studies included: first author, publication year, study aim, sample size, measurement tool, key study findings, conclusion, and funding source. In case of inaccessible material (such as lack of availability of full-text publication, missing data and/or uncertainties), the study authors were contacted for further information with a maximum of three attempts. 

### 2.6. Assessment of Methodological Quality

The quality assessment of studies followed the JBI Critical Appraisal Checklist for Analytical Cross-Sectional Studies [28] and assessed independently by two reviewers (Reima Mansour and Amit Arora). All studies were assessed and scored as ‘Yes’, ‘No’, ‘Unclear’, or ‘Not applicable’ against each of the eight questions (see Appendix D). Following the completion of the methodological quality assessment, disagreements (if any) between the two reviewers were resolved by discussion with the third author (Pranee Liamputtong) where necessary. Study authors were contacted in the event of insufficient details to confidently assess the methodological quality; and if a response was not received after three attempts, the study quality was assessed based on the available information.

## 3. Results

In the initial phase, a total of 1102 articles were identified across the selected electronic databases and by manual search. After the removal of duplicates, a total of 310 titles and abstracts were identified for further reading. Of these, five studies were identified for full-text reading. Three unsuccessful attempts were made to contact the authors of two of the studies to gain full text, so these studies were then also excluded (see Appendix C). Finally, three studies met the criteria and were included in the systematic review. A PRISMA flow diagram was constructed showing the identification, screening, eligibility, and included studies (Figure 1).

### 3.1. Methodological Quality

Each included study [13,29,30] demonstrated partially adequate methodological quality (see Appendix D). Overall, the criteria for inclusion in all studies were clearly defined with enough detail and all the necessary information critical to the study. The three included studies described the study sample in enough detail and the exposures were measured in a valid and reliable way. Three different tools were used to measure food security in the included studies. The use of different tools and sampling methods and sample sizes resulted in different levels of reliability and generalizability. The study by Dharod et al. [29] adjusted for potential confounders; however, the study by Alasagheirin and Clark [30] only ascertained the factors associated with food insecurity (using chi-square test). Anderson et al. [13] looked at association in their study; however, they did not reveal whether they controlled for confounding factors or not as the result for the interaction was not shown. The methods to assess outcome measures used by Anderson et al. [13] were not detailed. All included studies used validated instruments measurement tools and appropriate statistical analysis methods. 

### 3.2. Study Characteristics

Three articles were included in this systematic review. All three were quantitative cross-sectional studies. The publication dates ranged from 2013 to 2018. All three were published in English and had been conducted in USA, two among Sudanese migrants (including refugees) [13,30] and one among Somali refugee women [29]. Each study involved a structured, closed-answer questionnaire and one [30] also included bodily measurements and blood testing. 

Of the three USA studies included in the current review, the earliest (2013) identified their sample as Somali refugee women [29], while the second (2014) included recently arrived Sudanese refugee families [13]. The third, a 2018 study, interviewed North Sudanese refugee and migrant children between the ages of 5 and 18 years of age (assisted in their answers by their mother/caregiver or a research assistant) [30]. 

Dharod et al. [29] studied Somali mothers (who were primary household meal preparers) with at least one child 12 years or younger, resident in Lewiston, Maine. Participants (n = 195) were recruited using a snowball sampling method. Three bilingual interviewers contributed to the design of the questionnaire and were trained to administer it at the participants’ homes. The weight and height of each participant were also measured at the conclusion of the interview [29]. 

Anderson et al. [13] investigated Sudanese refugee families, surveying the family’s primary caregiver. Each family had at least one child under 3 years of age, and at least one legally resettled Sudanese parent who had been settled for 5 years or more. The study was conducted in a satellite town associated with Atlanta, Georgia. Participant recruitment used a snowball approach and began with a review of contacts in the case files of voluntary resettlement agencies working with Sudanese refugee families, the collection of names of volunteers at church and other community group meetings, and volunteers’ referrals being contacted by such volunteers. The interviewers were recruited by the lead researcher through local refugee outreach organizations and were trained for their role and actively contributed to the design of the survey. Ultimately, 49 of the 60 people surveyed completed their questionnaires [13]. 

Alasagheirin and Clark [30] conducted their study with Sudanese immigrant and refugee families in an unnamed Midwestern (USA) community. Potential participants (Sudanese migrant/refugee children aged between 5 and 18) were identified from a Sudanese society’s directory of families and were first contacted by a community leader regarding participation. Forty-seven families were thus identified as willing to participate and were contacted. Of these, 31 families, including 31 boys and 33 girls, participated. Participating families had resided in the USA from 1 to 14 years. Participant families were scheduled, as a unit, for an early morning appointment at the University of Iowa’s College of Nursing Research Suite to complete the surveys. After researchers had obtained informed consent, fasting blood samples were drawn and participants then were given a small breakfast. Children completed interviews and questionnaires with the assistance of a parent or a research assistant. Their body composition and other physical measurements were taken, and steps taken measured by a pedometer [30] (see Table 2).

### 3.3. Prevalence of Food Insecurity

All studies [13,29,30] cited a version of the USA Department of Agriculture’s definition for food security. Variations of different food security measurement tools were used to ascertain subjects’ food security status. One study used a version of the USDA Household Food Security Survey (HFSS) [30] while two used a modified form of the 12-item Radimer-Cornell Hunger Scale [13,29]. Indeed, Alasagheirin and Clark used only two items of the USDA HFSS 6-item short-form survey to measure food security [30], while Dharod et al. and Anderson et al. used the modified 10-item Radimer-Cornell Hunger Scale [13]. 

The rates of reported food insecurity were 40% [30], 67% [29], and 71% [13]. Anderson et al. found that not only did 71% experience some form of household food insecurity, 12% reported child hunger within the last month [13]. The rate of reported food insecurity for the populations studied was significantly higher than that for the general population in all of the three studies.

### 3.4. Determinants Associated with Food Insecurity

Anderson et al. [13] supplied a more nuanced picture of the practices of food insecure migrant families in the population they studied. They noted that while the consumption of many high-cost, nutrient-dense value foods (such as vegetables, fruit, meat, and dairy) generally decreased with increasing food insecurity status (a finding common in many such studies), the consumption (by adults) of high-cost, traditional food such as freshly killed meat (*p* = 0.049) was also linked to greater severity of food insecurity status. People in households determined by the research to be food insecure had a strong desire to serve traditional foods which they perceived as healthy (83%, 1.7 times more than those in food secure households (*p* = 0.014)) and important in the preservation of culture (78%, *p* = 0.005) among food insecure households [13]. 

While the study by Anderson et al. [13] did not find a relationship between the length of time caregivers had lived in the United States and household food insecurity, the study by Dharod et al. [29] found that recent arrivals (three years or less) were among those most affected by food insecurity as were those whose primary language in the home was Somali and those who reported limited education and lower English-speaking skills [29]. Alasagheirin and Clark [30], however, did not explore this relationship. 

### 3.5. Effects of Food Insecurity

Obesity and overweight were associated with food insecurity in the study by Dharod et al. [29]. They found that 41% of respondents had a body mass index (BMI) score, indicating overweight (25–29.9), and 24% had a BMI score of ≥30, indicating obesity. Food-insecure participants were almost 3 times more likely to be overweight or obese compared to food-secure women (OR: 2.66; CI: 1.25–-5.69; *p* = 0.01) [29]. 

A more complex picture was revealed in the 2018 study by Alasagheirin and Clark [30] where of the 64 children examined, 46% had a lean mass index (LMI) that was more than one standard deviation (SD) below the score expected based on a normal distribution. One-third of the children (32.7%) had very low bone mineral content (BMC). Over one-third (38%) had low spinal bone density (aBMD). According to the study, 21.8% of the children demonstrated wasting; and, depending upon the measurement used, the percentage of children overweight or obese compared to ‘American children’ varied. Using their BMI, 26.6% were overweight or obese (compared with the figure of 31.8% for other USA children), but when using a body fat percentage measure (BF%), 25.8% of boys and 30.3% of girls were obese compared to 19% and 20% of other USA boys and girls, respectively. Nearly one-fourth of children (23.4%) had either borderline or high cholesterol levels. Their physical activity results showed that no one over the age of 12 years reached the recommended 10,000 steps per day [30]. In contrast to other USA children, 40% of the children were food insecure [30]. Thus, food insecurity can be seen to have the potential to result in both underweight and overweight children, for both greater wasting and obesity among participants were revealed by this study. The detailed study also revealed the potential for long-lasting chronic illness that is associated with high cholesterol readings and low bone density. Circulatory and heart disease is associated with the former and, as youth is when the greatest proportion of adult bone density is accumulated, a lack of its accumulation in the early years can have grave ramifications in terms of fractures at an earlier age and those associated with osteopenia and osteoporosis in older age. Each of these medical conditions has earnings impacts for the sufferer as well as health costs for them and the community more generally.

## 4. Discussion

This systematic review identifies the prevalence, determinants, and effects of food insecurity among MENA migrants and refugees in HICs. Three studies met the inclusion criteria and were included in this systematic review [13,29,30]. All studies were conducted in USA, two among Sudanese migrant children or families [13,30], and one among Somali refugee women [29]. The rates of reported food insecurity ranged from 40% to 71% and were significantly higher than the general population. All three studies [13,29,30] showed that food insecurity had adverse health outcomes in migrants and refugees and noted that cultural norms, religion, and food preference play an important role in predicting food security and dietary habits of MENA migrants, including refugees. 

All three included studies [13,29,30] revealed a significantly high prevalence of food insecurity among MENA refugees and migrants in an HIC. These results are consistent with other studies [6,21] conducted in other HICs. In the UK, for example, a 2002 survey conducted among refugee families in East London found that all households sampled were food-insecure, and 60% of their children were experiencing hunger [21], while a 2018 systematic review by Lawlis et al. [6] reported food insecurity issues among refugees who had resettled in Australia. Framing food security in terms of food availability, access, utilization and stability, the 2018 review [6] described many factors associated with food insecurity, including cost and availability of traditional foods, difficulty accessing appropriate food outlets, limited food knowledge, low income, and lack of social support. Conducted among refugee groups (undifferentiated by ethnicity), the review reported that the prevalence of food insecurity varied from 35% to 90%, with severe hunger levels experienced by 11% to 40% of the participants [6]. 

Although USDA HFSS 18-item measure [31,32] is a highly sensitive and frequently utilized food security assessment tool, the studies by Dharod et al. [29] and Anderson et al. [13] elected to use the modified 10-item Radimer-Cornell Hunger Scale, while the study by Alasagheirin and Clark [30] used two items of the USDA HFSS 6-item short-form survey to measure food security. This variability in measurement tools could lead to some inconsistency in reporting the prevalence of food insecurity among studies and when comparing the study findings. Lawlis et al. [6] recommended, the adoption of a more rigorous measure of food insecurity than the currently used 2-item tool of the 2011–2012 Australian Health Survey which the authors believe may lead to underestimations of food insecurity. A study that included Sub-Saharan African migrants in Ottawa, Canada [25], echoed the findings of the three included studies in that almost half of the migrants were food insecure. This review found that food insecurity was most highly associated with their ethnicity (more than any other factor). This highlighted that a confluence of factors forms ethnically identified disadvantage. These include food availability, affordability, lower levels of migrant/refugee educational attainment (literacy and numeracy), recency of arrival (<5 years), reliance on social security, and lone motherhood [25]. The disadvantage created impedes food security. Again, it should be noted that food security is not just about having enough food to eat (that is, freedom from hunger), it should also be safe, nutritious, culturally acceptable, and obtained from a sustainable food system [6,7,9]. As the results of this review revealed, this is not always easily achievable. Many migrants (other than refugees) find themselves in a similar situation in HICs to a varying extent. Again, sample variations from refugees alone to a mixture of refugee and other migrant cohorts (such as business migrants, migrant under accepted employment schemes) or a failure to include participant income information in the data collected hamper comparison between groups, as well as with the general population or other subsets of population, and may contribute to some confounding of income impacts with ethnicity in relation to the causes of food insecurity. Many studies have noted that migrants (including refugees) generally have for some time been over-represented among those who endure higher levels of food insecurity. For example, a 2000 study by Kasper et al. investigated food insecurity among legal Latino and Asian immigrants (n = 630) and reported that 40% were food insecure without hunger and 41% were food insecure with hunger. Food insecurity was associated with low income, poor English, Latino ethnicity, and receipt of food assistance programs (‘food stamps’) [33]. Language difficulties can contribute to difficulty in securing employment and a lower than expected (or required) uptake of or participation in food assistance measures [34], but also to continued unemployment and poverty, and greater prevalence of developmental difficulties and chronic ill-health [35].

Two of the included studies [29,30] indicated a positive association between overweight and/obesity and food insecurity. This paradox has previously been confirmed in other groups, including US women, and Brazilian women and children, and the poor [36,37,38,39,40], but not among refugees and migrants as such. In the USA, for example, it has been found that it is neither ethnicity nor race that is the best predictor of obesity, but poverty [41]. Again, one study [30] found both wasting and obesity were over-represented among the sample population. This could lead to further detailed study to determine the factors (and their relative importance) that are most highly related to family/individual diet or lifestyle that produce such adverse dietary outcomes, factors such as opportunity (proximity of suitable food store, transport, location of fast food outlets, the ready availability of poorer nutritional quality foods), high cost of culturally appropriate foods, language, and income impact etc.

Alasagheirin and Clark [30] explored the impacts of food insecurity in greater depth than the other two included studies [13,29]. Other health impacts of food insecurity included bone density and body composition, poor skeletal growth, and higher metabolic risks [30]. Alasagheirin and Clark [30] noted that many children had transited through refugee camps in Egypt or Kenya, and deprivation in such situations could have affected growth and BMC to date, and these effects could be worsened by the observed low activity in the country of reception or compensated for (even if partly) by better nutrition and higher activity levels. As noted earlier, both high cholesterol and low bone mineral density have long term ramifications for those who continue to demonstrate such patterns [42,43].

The methodological quality of the included studies was partially adequate, with deficits found to have predominantly occurred due to the adoption of a non-probability sampling method. Furthermore, the findings cannot be generalized to the general population due to small sample sizes. Anderson et al. [13] used a cross-sectional study method instead of a prospective longitudinal study which led to an inability to make any causal inference. Although Dharod et al. [29] and Anderson et al. [13] used a snowball sampling technique which offers an advantage in accessing ‘hard to reach’ populations, this has limitations associated with the use of non-probability sampling techniques. None of the studies involved accessing a strictly representative sample. Authors of all three studies cited a small sample size (or smaller than desired sample size) as a limiting factor in analyzing and evaluating their research [13,29,30]. One study noted that a larger and more representative sample would be required to support their results [30]. Anderson and colleagues noted that a broader examination of cultural factors was needed for future research [13].

The current review had strengths and limitations. First, four databases were searched in order to gain a full collection of articles that reported on food security research among MENA migrants/refugees in high-income countries. Second, no limits were placed on publications in terms of date, age, gender, or language. Third, the included studies, whilst not comparable across the full range of their results, nevertheless added to available information related to recent MENA immigrants (including refugees) to HICs and their nutritional status (outlined above). 

Some limitations of this systematic review were noted, such as a failure to find the full texts of two studies that may be potentially relevant but were then excluded as we were unable to contact the authors despite repeated attempts. Secondly, a range of food security measurement tools other than the complete USDA Household Food Security Survey 18-item tool was used by included studies, making comparisons challenging. Dharod et al. [29] and Anderson et al. [13] used a modified 10-item Radimer-Cornell Hunger Scale, while Alasagheirin and Clark [30] used only two items of the USDA HFSS 6-item short-form survey. This variability of measurement tools could lead to some inconsistency when comparing the studies’ findings. 

## 5. Recommendations for Future Research 

Further research on food security among migrant populations, particularly those from Middle Eastern and North African communities, is warranted. There needs to be greater consideration of the qualitative, quantitative, and mixed-methods evidence on food security, diet, and nutrition among migrants in high-income countries. The methods for data collection should be comparable that rely on culture-specific language and food habits to ‘flesh out’ observations and support cogent interpretation. Furthermore, the origins and effects of food insecurity among the MENA population should be explored in greater detail to identify any unique characteristics (perhaps of cultural origin) that need to be addressed to facilitate greater food security. It would also be prudent to ascertain strategies adopted by migrants and refugees in their new environments in relation to food security and what could potentially be done to improve nutritional and health outcomes for MENA migrants and refugees [13,29]. Additionally, longitudinal studies could evaluate the longer-term impacts on the health of subjects from these recent migrant populations. More broadly on an international level, efforts from experts working in the area of food security should incorporate further input to develop a more comprehensive assessment of food insecurity to address this significant issue. 

## 6. Conclusions

This systematic review revealed that the prevalence of food insecurity is significantly high among MENA migrants and refugees. Food insecurity adversely impacts the health (e.g., low bone mass, low muscle mass, and high percentage of body fat) of MENA migrants and refugees. Food insecurity was associated with a number of factors, including the degree of acculturation and socio-demographic factors. However, while the three included studies examined socio-demographic factors, the degree of acculturation was largely unexplored other than in terms of length of residence. Further research on food security among the MENA migrant population, its origins, and its effects is warranted to address this public health issue. 

## Figures and Tables

**Table 1:** Eligibility criteria according to SPIDER ^1^ criteria.

SPIDER Tool	Search Terms
S	Middle Eastern or North African migrants or refugees residing in a high-income country
P and I	Studies investigating the status of food security and challenges related to migrant/refugee access to affordable foods that meet their cultural needs.
D	No specific study design
E	Views and experiences of the members of participant groups
R	Both qualitative and quantitative

^1^ Sample (S), Phenomenon of Interest (PI), Design(D), Evaluation(E), Research type(R).

**Table 2:** Literature review of the included studies (n = 3).

Citation	Study Design			Study Findings	Conclusion	Source of Funding
Author(s)/Year	Participants/Eligibility	Sampling/Recruitment/Study Setting	Data Collection/Tool	Demographic/FS/FS Impacts		
Dharod et al. 2013 [29]	Eligibility: Women resident of Lewiston, Maine (US); have at least 1 child and the main meal preparer of the household. Ethnicity/Nationality: Somali	Method: Cross-sectional/convenience sample Size: 195 Somali women Recruitment: using a snowball sampling method Study setting: Lewiston (Maine)	Ten-item Radimer/Cornell Hunger Scale (Questionnaire)	Food Insecure (n = 131) Food Secure (n = 64) 3 times more likely to be overweight or obese	Somali refugees experienced high levels of FIS upon resettlement. Poor dietary habits and the high overweight/obesity rate among insecure families call for future research in understanding what role family structure, cultural norms, and food preference play in predicting food security and dietary habits among Somali and overall African refugees in the US.	Not reported
Anderson et al. (2014) [13]	Eligibility: Each family had at least one child under 3, and at least one legally resettled Sudanese parent who had been resettled for at least 5 years. Ethnicity/Nationality: Sudanese refugee families	Method: Cross-sectional Size: 49 recently arrived refugees. Recruitment: recruited through voluntary resettlement agency (VOLAG) case lists, church and community groups, and word-of-mouth (snowball approach) Study setting: Metropolitan Atlanta, USA	10-item modified version of the Radimer/Cornell hunger scale (Questionnaire)	71% experienced some form of household food insecurity:12% reported child hunger FIS associated with more frequent consumption of some low-cost, traditional Sudanese foods.	Increasing severity of household FIS was associated with decreased consumption of high-cost, high-nutrient-density food items.	Emory University Research Committee, and the Office of University-Community Partnerships of Emory University provided financial and in-kind support for data collection. Analysis was funded by awards from the Canada Research Chairs program (DWS) and the Canadian Institutes for Health Research (LA).
Alasagheirin and Clark, 2018 [30]	Eligibility: Families with children (5 and 18 years old) and had lived in the United States Ethnicity/Nationality: Northern Sudan, Muslim and spoke Arabic	Method: Cross- sectional study Size: 31 families, including 64 children, 31 boys, and 33 girls. Recruitment: Identified from a Sudanese Society’s directory of families and were first contacted by a community leader regarding participation. Study setting: Midwestern satellite town of the Atlanta conurbation	Two questions from the U.S. Household Food Security Survey Module (Questionnaire + bodily measurements and blood testing)	Food insecurity 40% of families 26.6% were overweight or obese	Sudanese children may have unique risks related to low bone mass low muscle mass, high percent body fat metabolic biomarkers, inactivity, and FIS potentially contributing to adult osteoporosis, diabetes, and cardiovascular disease.	The Institute for Clinical and Translational Science at the University of Iowa (CTSA) program, grant UL1 TR000442.

## Appendix A

**Table A1:** PRISMA checklist.

Section/Topic	Item no.	Checklist Item	Reported on Page no.
**TITLE**			
Title	1	Identify the report as a systematic review, meta-analysis, or both.	1
**ABSTRACT**			
Structured summary	2	Provide a structured summary including, as applicable: background; objectives; data sources; study eligibility criteria, participants, and interventions; study appraisal and synthesis methods; results; limitations; conclusions and implications of key findings; systematic review registration number.	1
**INTRODUCTION**			
Rationale	3	Describe the rationale for the review in the context of what is already known.	2, 3
Objectives	4	Provide an explicit statement of questions being addressed with reference to participants, interventions, comparisons, outcomes, and study design (PICOS).	3
**METHODS**			
Protocol and registration	5	Indicate if a review protocol exists, if and where it can be accessed (e.g., Web address), and, if available, provide registration information including registration number.	NA
Eligibility criteria	6	Specify study characteristics (e.g., PICOS, length of follow-up) and report characteristics (e.g., years considered, language, publication status) used as criteria for eligibility, giving rationale.	3
Information sources	7	Describe all information sources (e.g., databases with dates of coverage, contact with study authors to identify additional studies) in the search and date last searched.	3
Search	8	Present full electronic search strategy for at least one database, including any limits used, such that it could be repeated.	3
Study selection	9	State the process for selecting studies (i.e., screening, eligibility, included in systematic review, and, if applicable, included in the meta-analysis).	4
Data collection process	10	Describe method of data extraction from reports (e.g., piloted forms, independently, in duplicate) and any processes for obtaining and confirming data from investigators.	4
Data items	11	List and define all variables for which data were sought (e.g., PICOS, funding sources) and any assumptions and simplifications made.	3
Risk of bias in individual studies	12	Describe methods used for assessing risk of bias of individual studies (including specification of whether this was done at the study or outcome level), and how this information is to be used in any data synthesis.	N/A (Cross Sectional)
Summary measures	13	State the principal summary measures (e.g., risk ratio, difference in means).	4
Synthesis of results	14	Describe the methods of handling data and combining results of studies, if done, including measures of consistency (e.g., I2) for each meta-analysis.	4
Risk of bias across studies	15	Specify any assessment of risk of bias that may affect the cumulative evidence (e.g., publication bias, selective reporting within studies).	N/A (Cross Sectional)
Additional analyses	16	Describe methods of additional analyses (e.g., sensitivity or subgroup analyses, meta-regression), if done, indicating which were pre-specified.	N/A
**RESULTS**			
Study selection	17	Give numbers of studies screened, assessed for eligibility, and included in the review, with reasons for exclusions at each stage, ideally with a flow diagram.	6
Study characteristics	18	For each study, present characteristics for which data were extracted (e.g., study size, PICOS, follow-up period) and provide the citations.	6
Risk of bias within studies	19	Present data on risk of bias of each study and, if available, any outcome level assessment (see item 12).	N/A
Results of individual studies	20	For all outcomes considered (benefits or harms), present, for each study: (a) simple summary data for each intervention group (b) effect estimates and confidence intervals, ideally with a forest plot.	9
Synthesis of results	21	Present results of each meta-analysis done, including confidence intervals and measures of consistency.	7, 8
Risk of bias across studies	22	Present results of any assessment of risk of bias across studies (see Item 15).	N/A
Additional analysis	23	Give results of additional analyses, if done (e.g., sensitivity or subgroup analyses, meta-regression [see Item 16]).	N/A
**DISCUSSION**			
Summary of evidence	24	Summarize the main findings including the strength of evidence for each main outcome; consider their relevance to key groups (e.g., healthcare providers, users, and policy makers).	12
Limitations	25	Discuss limitations at study and outcome level (e.g., risk of bias), and at review-level (e.g., incomplete retrieval of identified research, reporting bias).	12
Conclusions	26	Provide a general interpretation of the results in the context of other evidence, and implications for future research.	13
**FUNDING**			
Funding	27	Describe sources of funding for the systematic review and other support (e.g., supply of data); role of funders for the systematic review.	13

## Appendix B

**Table A2:** CINAHL search–Ovid interface.

S	MeSH Terms	Output
S1	(MH “Food Security”) OR “food security”	4253
S2	(“MH Food insecurity”) OR “food insecurity”	3641
S3	(MH “Food Preferences”)	6201
S4	“Food access” OR “Food accessibility”	431
S5	“Food stability”	89
S6	“Food availability”	1556
S7	(MH “Refugees”) OR “refugees”	8208
S8	“displaced persons” OR “resettled”	5862
S9	(MH “Immigrants”) OR “immigrants” OR migrant	24,601
S10	S1 OR S2	5287
S11	S3 OR S4 OR S5 OR S6	8041
S12	S7 OR S8 OR S9	31,601
S13	S10 OR S11	12,888
S14	S12 AND S13	296

## Appendix C

**Table A3:** Reasons for Excluded Studies.

Author(s) and Year (Reference Number)	Title	Reasons for Exclusion
Bertmann et al. 2016	A Pilot Study of Food Security among Syrian Refugees in Schleswig-Holstein, Germany.	Not enough data available
Ebadi et al. 2017	Food Security and International Migration: A comparative study of Asia, Middle East/North Africa, Latin America/Caribbean and Sub-Saharan Africa.	Not enough data available

## Appendix D

**Table A4:** JBI Critical Appraisal Checklist for Analytical Cross-Sectional Studies.

JBI Critical Appraisal	Dharod & Clark, 2013 [29]	Anderson et al. (2014) [13]	Alasagheirin et al., 2018 [30]	Comments
Were the criteria for inclusion in the sample clearly defined?	Yes	Yes	Yes	
Were the study subjects and the setting described in detail?	Yes	Yes	Yes	
Was the exposure measured in a valid and reliable way?	Yes	Yes	Yes	
Were objective, standard criteria used for measurement of the condition?	Yes	Yes	Yes	Three different tools were used to measure food security (see Discussion above)
Were confounding factors identified?	Yes	Unclear	No	
Were strategies to deal with confounding factors stated?	No	No	No	
Were the outcomes measured in a valid and reliable way?	Yes	No	Yes	The use of different tools and sampling methods and sample size resulted in different levels of reliability, generalizability.
Was appropriate statistical analysis used?	Yes	Yes	Yes	
